# Cytology–Based Specimen Triage for Epidermal Growth Factor Receptor Mutation Testing of Malignant Pleural Effusions in Non–Small Cell Lung Cancer

**DOI:** 10.3389/fonc.2022.810124

**Published:** 2022-01-24

**Authors:** Chi-Lu Chiang, Chia-I Shen, Hsu-Ching Huang, Han-Jhih Chang, Yu-Ting Huang, Chao-Hua Chiu

**Affiliations:** ^1^ Department of Chest Medicine, Taipei Veterans General Hospital, Taipei, Taiwan; ^2^ School of Medicine, National Yang Ming Chiao Tung University, Taipei, Taiwan; ^3^ Institute of Clinical Medicine, National Yang Ming Chiao Tung University, Taipei, Taiwan

**Keywords:** cell–free DNA, cytology, epidermal growth factor receptor, non–small cell lung cancer, pleural effusion

## Abstract

**Introduction:**

Malignant pleural effusions are common in non–small cell lung cancer (NSCLC). Molecular testing is among the most critical steps in the management of patients with advanced NSCLC. However, the optimal approach for epidermal growth factor receptor (EGFR) mutation testing in such effusion samples remains unclear.

**Methods:**

We prospectively collected effusion samples from patients with EGFR–mutant NSCLC. Following sample centrifugation, genomic DNA and cell–free DNA were respectively extracted from the sediment and supernatants. EGFR mutation was detected through a real–time PCR assay.

**Results:**

A total of 108 effusions from 78 patients were examined, with 12 and 96 obtained before and after EGFR tyrosine kinase inhibitor treatment, respectively. Carcinoma cells or atypical cells were identified in 73 effusions (67.6%). EGFR mutations were detected in 86 (79.6%) sediment and 84 (77.8%) supernatant samples. Among the effusions with positive cytological findings, the EGFR mutation detection rates were 95.9% (70/73) and 86.3% (63/73) in the sediment and supernatants, respectively. Among the effusions with negative cytological findings, the corresponding detection rates were 45.7% (16/35) and 60% (21/35), respectively. Current clinical practice is to arrange EGFR mutation testing only for sediment from cytologically positive effusions. Through the proposed cytology–based specimen triage, wherein sediment and supernatants with positive and negative cytological findings, respectively, are tested, the detection rate was increased from 64.8% (70/108) to 84.3% (91/108). At half of the cost, this strategy provided a detection rate only slightly lower than the rate in a combined test of all the sediment and supernatants (87.0%, 94/108).

**Conclusions:**

The separate extraction of DNA from sediment and supernatants obtained from centrifuged effusion samples can improve the overall EGFR mutation detection rate. The present cytology–based specimen triage is an efficient strategy for EGFR mutation testing in patients with EGFR–mutant NSCLC.

## Introduction

Molecular profiling of tumors for driver mutation detection has become a standard of care for several types of advanced malignancies, particularly non–small cell lung cancer (NSCLC) (1). Several studies have demonstrated that epidermal growth factor receptor (EGFR) tyrosine kinase inhibitor (EGFR–TKI) treatment of patients with advanced EGFR–mutant NSCLC yields superior survival outcomes to chemotherapy (2–4). Extraction of tumor tissue for oncogene detection is routine in clinical practice; however, obtaining sufficient quantities for molecular analysis is sometimes challenging. Malignant pleural effusions (MPEs) are often observed in NSCLC, especially adenocarcinomas, which are typically located in the periphery and invade the pleura (5). Studies have indicated that MPE samples can be used as surrogates for lung tumor samples for EGFR mutation detection, and that the results are correlated with response to EGFR–TKI treatment (6–8).

The DNA used for analyzing mutations in MPE samples is typically extracted from malignant cells. The cytology positive rate of MPE in NSCLC patients was around 80% (9). The amount or the percentage of tumor cells is sometimes insufficient for mutation analysis. In the past, supernatants from MPE centrifugation were discarded. Recent analyses of cell–free DNA (cfDNA) in supernatants have revealed promising results in the detection of EGFR mutation, even in cytologically negative cases (10–13). However, given the between-study variability in platforms used for DNA sequencing, the utility of supernatants from MPE samples in EGFR mutation analysis remains questionable. The correlation between cytological abnormalities and EGFR mutation detection rates in MPE sediment and supernatants warrants further investigation.

The present study evaluated the clinical value of testing both sediment and supernatant from MPE samples with a commercially available EGFR mutation assay kit. We developed an efficient approach for optimizing the detection of EGFR mutation in the MPE samples of patients with NSCLC.

## Materials and Methods

### Patients and Sample Preparation

**Figure 1:**
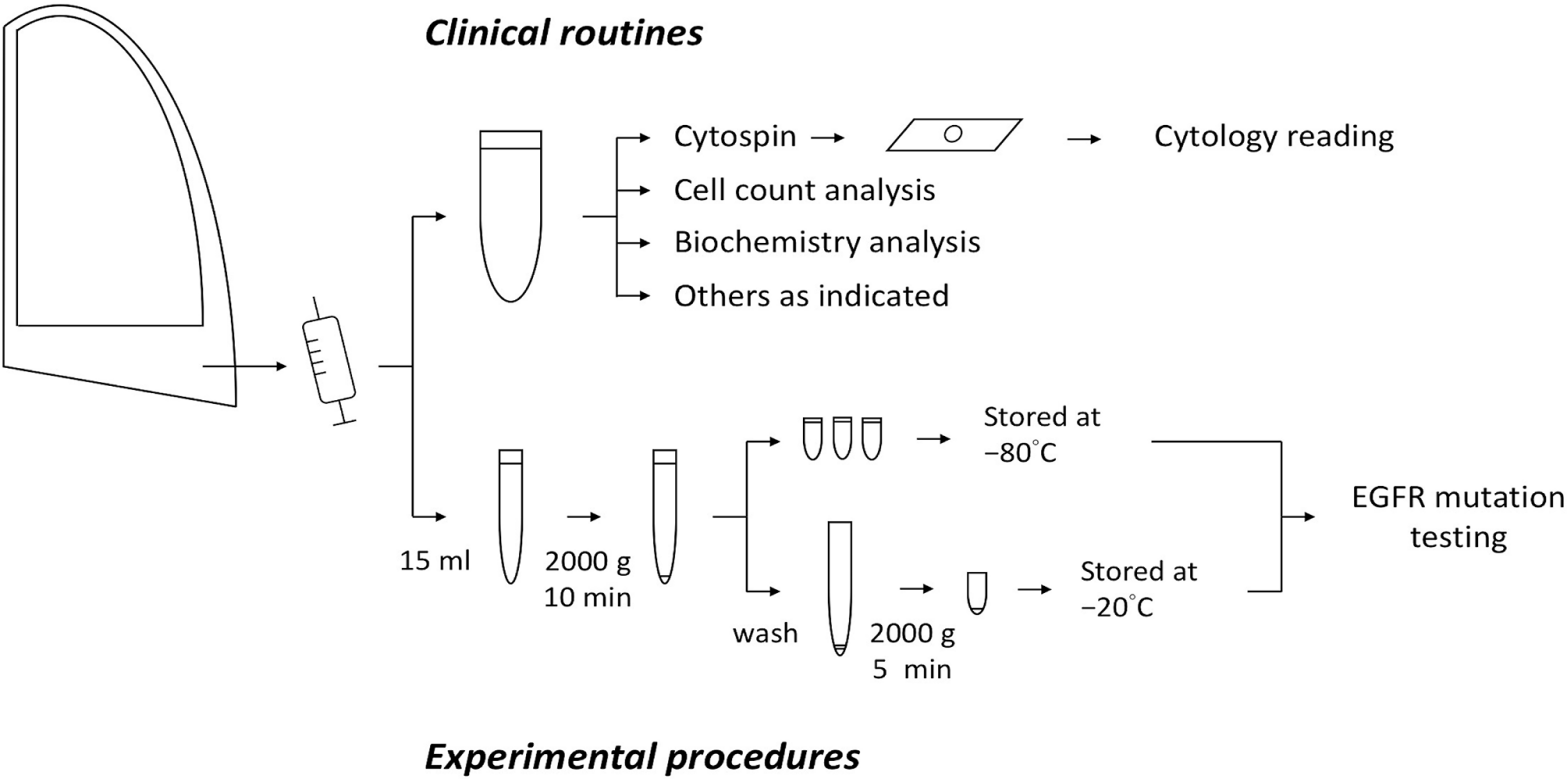
The experimental procedures in this study.

The participants comprised patients who had EGFR–mutant NSCLC with substantial body effusions requiring diagnostic tapping or drainage for symptom relief. Effusion samples in standard amounts were sent for routine clinical laboratory tests, including cytological examination. Fifteen milliliter of the residual effusions were collected for further analysis. After the first round of centrifugation at 2000 ×g for 10 min, the supernatant was collected, aliquoted, and stored at −80°C until cfDNA extraction. The crude sediment was washed with phosphate-buffered saline and centrifuged at 2000 ×g for 5 min, after which it was collected and stored at −20°C until DNA extraction. All samples were processed at room temperature within 1 hour of sample collection. The preparation procedures are illustrated in **Figure 1**. The study protocol was approved by the institutional review board and all the patients provided written informed consent.

### Cytological Examination

Cytological procedures and readings were performed as clinical routine assessments. In brief, cytospin preparations were made using the cytocentrifuge at 400 ×g for 5 min at room temperature. Two smears were prepared and were subjected to Papanicolaou and Liu’s staining for morphological assessment. The cytological examinations were performed by 2 board-certified cytologists and were reported as the presence of carcinoma cells, the presence of atypical cells, or the absence of malignant cells. Each sample was also categorized according to its total cellularity (scant, moderate, or abundant) and the percentage of carcinoma or atypical cells (0%, < 5%, 5% to 49%, or ≥ 50%).

### DNA Extraction

The frozen sediment of each effusion was resuspended in 200 μL of phosphate-buffered saline. Genomic DNA was extracted using the High Pure PCR Template Preparation Kit (Roche Diagnostics, Mannheim, Germany) according to the manufacturer’s instructions. Isolated DNA was quantified using the NanoDrop Lite Spectrophotometer (Thermo Fisher Scientific, Wilmington, DE, USA), and a working sample solution with a final concentration of 4 ng/μL was prepared. cfDNA was extracted from the supernatant by using the using QIAamp Circulating Nucleic Acid Kit (Qiagen, Hilden, Germany) according to the manufacturer’s instructions. It was isolated using 4 mL of supernatant (starting volume). This was followed by elution in 180 µL of elution buffer.

### EGFR Mutation Testing

EGFR mutation testing was performed and interpreted as previously described (14). In brief, the extracted DNA was analyzed using the cobas EGFR Mutation Test v2 (Roche Diagnostics, Branchburg, NJ, USA). The test is based on a mutant allele-specific, real-time PCR-based, mutation detection technology designed to identify 42 mutations in exons 18 to 21 of EGFR. For each sample, 75 μL of sample solution was examined under the cobas z 480 analyzer for automated amplification and detection. The final results are presented as “mutation detected”, “mutation undetected”, or “invalid”.

### Statistical Analysis

The associations between the patient and/or sample characteristics were analyzed using the Pearson chi-square test, and the associated *P* values were 2 sided. Fisher’s exact test was performed when one or more cells contained fewer than 5 observations, and linear-by-linear association was applied to ordinal variables. The Student’s t test was used for the comparison of DNA concentrations between 2 groups. Analyses were conducted using IBM SPSS Statistics for Windows, version 20 (IBM Corp., Armonk, NY, USA).

## Results

### Patient Characteristics

From December 2017 through July 2020, a total of 108 effusion samples from 78 patients with advanced NSCLC were prospectively collected and EGFR mutation testing was performed within three months after collection (median, 8.5 days; range, 0 – 91 days). The patient characteristics are summarized in **Table 1**. Overall, 47 (60.3%) patients were female, 68 (87.2%) were never-smokers, and the median age was 66 years. The patients’ EGFR mutation statuses at initial presentation are summarized as follows: EGFR exon 19 deletion was detected in 39 (50.0%) patients, exon 21 L858R substitution was detected in 30 (38.5%) patients, 4 (5.1%) had coexisting *de novo* T790M mutations, and 5 (6.4%) had uncommon mutations (G719X and L861Q). In total, 54 patients contributed one effusion sample each, whereas 19, 4, and 1 patient provided 2, 3, and 4 samples in different clinical scenarios at different time points, respectively.

**Table 1:** Patient characteristics (N = 78).

	N	(%)
Gender		
Female	47	60.3
Male	31	39.7
Age		
Median	66	
Range	37 – 90	
Smoking		
Never	68	87.2
Ever	10	12.8
Histology		
Adenocarcinoma	77	98.7
NSCLC–NOS	1	1.3
Staging at initial diagnosis		
I	6	7.7
II	1	1.3
III	12	15.4
IV	59	75.6
EGFR mutation at diagnosis		
Exon 19 deletion	39	50.0
L858R	30	38.5
G719X	3	3.8
L861Q	2	2.6
Exon 19 deletion + T790M	1	1.3
L858R + T790M	3	3.8
Effusion sampling		
1 time	54	69.2
2 times	19	24.4
3 times	4	5.1
4 times	1	1.3

NSCLC–NOS, non–small cell lung cancer–not otherwise specified.

### Effusion Characteristics

Of the 108 effusion samples, 103 (95.4%) were pleural effusions and 5 (4.6%) were ascites, and 12, 87, and 9 samples (11.1%, 80.6%, and 8.3%) were obtained before EGFR–TKI treatment, after 1 EGFR–TKI treatment session, and after 2 or more EGFR–TKI treatment failures, respectively. The most recent treatment that 57 and 39 patients (52.8% and 36.1%, respectively) received was EGFR–TKI and chemotherapy, respectively. Regarding the remaining 12 patients, 9 (8.3%) were treatment naïve and 3 (2.8%) had not received cancer treatment in more than 3 months. The cellularity of the effusions was mostly moderate to abundant (67.6%). Carcinoma cells or atypical cells were identified in 73 (67.6%) samples. The proportions of carcinoma cells or atypical cells were < 5%, 5% to 49%, and ≥ 50% in 41, 17, and 15 samples, respectively. The details are summarized in **Table 2**.

**Table 2:** Effusion characteristics (N = 108).

	N	(%)
Sample type		
Pleural effusion	103	95.4
Ascites	5	4.6
Cellularity		
Scant	33	30.6
Moderate	45	41.7
Abundant	30	27.8
Cytology		
Negative for malignant cells	35	32.4
Carcinoma or atypical cells	73	67.6
Carcinoma or atypical cell percentage		
0%	35	32.4
<5%	41	38.0
5 – 49%	17	15.7
≥50%	15	13.9
Pellet DNA concentration (ng/μL)		
Mean	353.0	
Range	13.3 – 1724.0	
EGFR–TKI exposure history		
EGFR–TKI naïve	12	11.1
First generation EGFR–TKI	52	48.1
Second generation EGFR–TKI	34	31.5
Third generation EGFR–TKI	1	0.9
First and second generation EGFR–TKI	2	1.9
First/second and third generation EGFR–TKI	7	6.5
Last treatment before sampling		
Treatment naïve	9	8.3
First/second generation EGFR–TKI	49	45.4
Third generation EGFR–TKI	8	7.4
Chemotherapy	39	36.1
Treatment-free > 3 months	3	2.8

EGFR–TKI, epidermal growth factor receptor-tyrosine kinase inhibitor.

In 108 sediment samples, the mean DNA concentration was 353.0 ng/μL. The concentration was significantly lower in samples with scant cellularity than in those with moderate-to-abundant cellularity (261.6 ng/μL vs. 393.2 ng/μL, *P* = 0.028). Moreover, the mean DNA concentration in cytologically negative samples was significantly lower than in samples containing carcinoma cells or atypical cells (158.8 ng/μL vs. 446.1 ng/μL, *P* < 0.001).

### EGFR Mutation Testing

Two of the 108 sediment samples (1.9%) yielded invalid results. EGFR mutations were detected in 86 (79.6%) samples. Detection rates in samples with cytologically positive findings and samples with cytologically negative findings were 95.9% (70/73) and 45.7% (16/35), respectively (**Table 3A**). Eight of the 108 supernatant samples (7.4%) yielded invalid results. EGFR mutations were detected in 84 (77.8%) samples. Detection rates in in samples with cytologically positive findings and samples with cytologically negative findings were 86.3% (63/73) and 60.0% (21/35), respectively (**Table 3B**).

**Table 3:** Effusion cytology and EGFR mutation status.

(A) By sediments								
Cytology	EGFR mutation testing result						N	
	Invalid		Mutation undetected		Mutation detected			
Negative	1	2.9%	18	51.4%	16	45.7%	35	100.0%
Positive	1	1.4%	2	2.7%	70	95.9%	73	100.0%
	2	1.9%	20	18.5%	86	79.6%	108	100.0%
**(B) By supernatants**								
**Cytology**	**EGFR mutation testing result**						**N**	
	**Invalid**		**Mutation undetected**		**Mutation detected**			
Negative	3	8.6%	11	31.4%	21	60.0%	35	100.0%
Positive	5	6.8%	5	6.8%	63	86.3%	73	100.0%
	8	7.4%	16	14.8%	84	77.8%	108	100.0%

**Figure 2:**
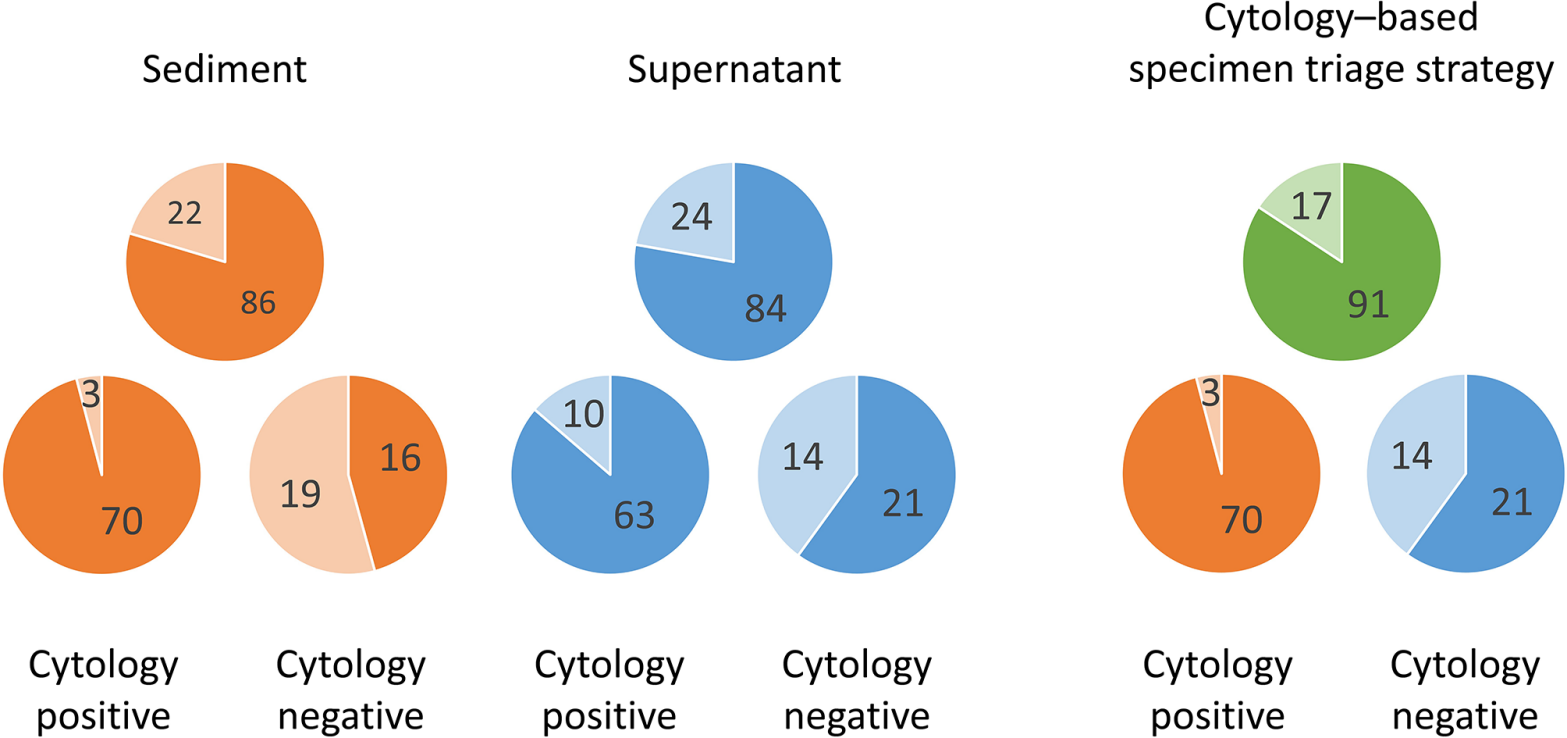
Differences of EGFR mutation detection rates in effusion sediments and supernatants by cytology examination status (positive, n = 73; negative, n = 35) and the advantage of the proposed cytology–based specimen triage strategy. Darker color, mutation detected; lighter color, mutation undetected.

In terms of paired sediment and supernatant samples, none showed both invalid results. EGFR mutations were detected in both sediment and supernatants in 76 samples (70.4%), in sediment only in 10 samples (9.3%), in supernatants only in 8 samples (7.4%), and in neither sediment nor supernatants in 14 samples (13.0%). Overall, the detection rate increased from 79.6% (86/108) in the sediment testing to 87.0% (94/108) in the testing of both sediment and supernatants (**Table 4A**). In samples with cytologically positive findings, the detection rate was higher in the sediment (95.9%, 70/7) than in the supernatants (86.3%, 63/73) (**Table 4B**). However, in the cytologically negative samples, the detection rate was higher in the supernatants (60%, 21/35) than in the sediment (45.7%, 16/35) (**Table 4C**). If one considered the current clinical practice as the standard, i.e. only sent the sediment of the cytologically positive effusion samples for EGFR mutation testing, the detection rate was 64.8% (70/108) in this cohort. When we triaged the specimens for EGFR mutation testing, i.e. using the sediment from the cytologically positive samples and the supernatants from the cytologically negative samples, the detection rate significantly increased to 84.3% (91/108) (*P* < 0.001) (**Figure 2**). It was only slightly lower than the detection rate in a combined test of all the sediment and supernatants (87.0%, 94/108).

**Table 4:** EGFR mutation status by sediments and supernatants by cytology.

(A) All samples				
Sediments	Supernatants			N
	Invalid	Mutation undetected	Mutation detected	
Invalid	0	0	2	2
Mutation undetected	2	12	6	20
Mutation detected	6	4	76	86
	8	16	84	108
**(B) Samples with positive cytology**				
**Sediments**	**Supernatants**			**N**
	**Invalid**	**Mutation undetected**	**Mutation detected**	
Invalid	0	0	1	1
Mutation undetected	0	2	0	2
Mutation detected	5	3	62	70
	5	5	63	73
**(C) Samples with negative cytology**				
**Sediments**	**Supernatants**			**N**
	**Invalid**	**Mutation undetected**	**Mutation detected**	
Invalid	0	0	1	1
Mutation undetected	2	10	6	18
Mutation detected	1	1	14	16
	3	11	21	35

In the 88 sediment samples obtained from patients resistant to first- or second-generation EGFR–TKIs, 68 were positive for EGFR mutations. Specifically, 44 were positive for sensitive EGFR mutations alone and 24 were positive for both sensitive and T790M mutations. In the supernatant samples, 68 tested positive for EGFR mutations. Specifically, 39 were positive for sensitive EGFR mutations alone, 28 were positive for both sensitive and T790M mutations, and 1 was positive only for the T790M mutation (**Table 5**). Combining the testing results for both the sediment and supernatants, T790M mutation was identified in 32 of 75 effusion samples that tested positive for EGFR mutations.

**Table 5:** EGFR T790M mutation status by sediments and supernatants.

Sediments	Supernatants					N
	Invalid	Mutation undetected	Sen. Mutation + T790M	Sen. Mutation alone	T790M alone	
Invalid	0	0	1	1	0	2
Mutation undetected	2	11	0	5	0	18
Sen. Mutation + T790M	1	0	21	2	0	24
Sen. Mutation alone	2	4	6	31	1	44
T790M alone	0	0	0	0	0	0
	5	15	28	39	1	88

Sen., Sensitive.

## Discussion

Testing for tumor genomic alterations by using peripheral blood samples, commonly referred to as liquid biopsy, can be performed as a surrogate for tissue molecular testing and can be observed in recent clinical use (15). However, there are liquids other than blood in human body, for example, pleural effusion which is frequently encountered in lung cancer patients. After centrifugation, clinicians typically subject the cell pellets from the effusions to DNA extraction and molecular testing and discard the supernatants as medical waste. In the present study, we evaluated the usefulness of effusion supernatants as a medium for EGFR mutation testing in patients with EGFR–mutant NSCLC. High detection rates were observed, and the rates in the sediment and supernatants were comparable. Notably, testing of supernatants from cytologically negative effusions yielded a mutation detection rate as high as 60% in these samples that are typically not subjected to molecular testing. Furthermore, resistance mutations were detected in the effusions of patients who had undergone EGFR–TKI treatment. The results suggest that effusions can be used for disease monitoring and treatment guidance in relapse.

Liquid biopsy *via* plasma has become a practical alternative source for genetic testing in patients with advanced NSCLC and could be used concurrently or sequentially to tissue genotyping in clinical practice (16). As does plasma, effusion supernatants contain abundant cfDNA and thus can facilitate clinical diagnosis. In this study, we demonstrated that EGFR mutations were detected in cfDNA from supernatants in 77.8% of the samples, comparable to the detection rate corresponding to the genomic DNA from the sediment (79.6%). Kimura et al. detected EGFR mutations in 11 of 43 supernatants from pleural effusion samples through direct sequencing (17). Using a methodology similar to ours, Liu et al. used tissue as a standard and reported sensitivities of 63.6% in supernatants and 81.8% in cell blocks (18). Similar results have also been reported in multiple other studies (10, 11, 19–23).

Pleural metastasis is not always associated with malignant cells in effusions (24). One study indicated that EGFR mutations are not detectable from cytologically negative effusions (17). However, recent studies have demonstrated that they are detectable when a sensitive method is used (11, 13). Detection rates are clearly associated with positive cytological findings. We detected EGFR mutations in 95.9% of the sediment samples from the cytologically positive effusions but only in 45.7% of the sediment samples from the cytologically negative effusions. Notably, the difference was smaller in the supernatants (86.3% vs. 60%), in line with the findings of Kawahara et al. (11). The failure of cytological examination to detect malignant cells has several possible explanations, including there is low tumor burden, cancer cells cannot survive in effusions, and cancer cells do not detach from the pleura. The situations in these explanations presumably result in the presence of few or no cancer cells in sediment, thus yielding false-negative results for EGFR mutations. However, cfDNA could be either actively secreted by living cancer cells on the pleura surface or passively released from necrotic cancer cells in effusions. Therefore, the rate of EGFR mutation from the supernatants is less likely to be influenced by negative cytological findings.

The optimal strategy for EGFR mutation testing in a given clinical scenario invariably entails the joint considerations of sensitivity, turnaround time, and cost. Typically, in current clinical practice, only the sediment samples of the cytologically positive effusions are tested for EGFR mutations. This would mean that 70 of the 73 cytologically positive samples in this study would test positive for EGFR mutations, corresponding to an overall sensitivity of 64.8%. Combined testing of the sediment and supernatants, irrespective of cytology results, yielded the highest sensitivity rate (87.0%) and shortest turnaround time but doubled the cost. On the basis of our findings, we propose a cytology–based specimen triage strategy wherein sediment from cytologically positive effusions and supernatants from cytologically negative effusions are separately subjected to EGFR mutation testing. Through this approach, EGFR mutations would be detectable in 91 (84.3%) of the 108 samples. This approach results in considerably higher sensitivity with the same cost as that in the conventional approach and half the cost of the combined test, with only a slight reduction in sensitivity. We believe that the proposed approach can optimize the detection rate of EGFR mutation in effusion samples from patients with NSCLC.

Rebiopsy after acquired resistance to targeted therapy can guide subsequent treatment by providing insights into histological or genetic changes (25, 26). The secondary EGFR T790M mutation is the most common mechanism of resistance to first- or second-generation EGFR–TKIs, accounting for the resistance in approximately 52.8% of Taiwanese patients with NSCLC (27). Detection of the T790M mutation by assessing circulating cfDNA has clinical potential. In the present study, the rate of T790M mutation detection from the sediment or supernatants was comparable, and the overall T790M detection rate was 42.6%. These results are consistent with those from previous studies, further confirming that effusion or other body fluids can be a valuable source for mutation analysis in case of acquired resistance to targeted therapy (12, 28).

This study has some limitations. First, we did not analyze other genetic alterations in the effusion samples; therefore, we could not construct a comprehensive mutation profile. Next-generation sequencing had been reported to be feasible using cytology specimens from pleural effusions. It not only can evaluate genetic aberrations comprehensively but also has greater sensitivity than PCR-based assays (29, 30). However, this novel sequencing platform is still not clinically available worldwide. Osimertinib, a third-generation EGFR–TKI overcoming T790M resistant mutation, had become a new treatment opinion in the first line setting. However, the resistance mechanism to osimertinib cannot be investigated in our study since most patients received first- or second-generation EGFR–TKI as front-line treatment. Second, although we confirmed that EGFR mutations are detectable in supernatants, the association of these detection rates with response to EGFR–TKI treatment remains unclear. Further studies employing methods with higher sensitivity and comprehensiveness may be warranted to investigate the evolution of driver mutations detectable in malignant effusions in patients with NSCLC.

In conclusion, the separate extraction of DNA from sediment and supernatants obtained from centrifuged effusion samples can improve the overall detection rate of EGFR mutations. The present cytology–based specimen triage approach is an efficient strategy for EGFR mutation testing of malignant effusions in patients with EGFR–mutant NSCLC.

## Data Availability Statement

The original contributions presented in the study are included in the article/supplementary material. Further inquiries can be directed to the corresponding author.

## Ethics Statement

The studies involving human participants were reviewed and approved by the Institutional Review Board, Taipei Veterans General Hospital. The patients/participants provided their written informed consent to participate in this study.

## Author Contributions

C-LC: Conceptualization, Investigation, Formal analysis, Writing—original draft. C-IS: Investigation, Validation. H-CH: Investigation, Validation. H-JC: Investigation, Resources. Y-TH: Investigation, Resources. C-HC: Conceptualization, Investigation, Formal analysis, Validation, Resources, Writing—Review and Editing, Supervision, Project administration, Funding acquisition. All authors contributed to the article and approved the submitted version.

## Funding

This work was supported by the Ministry of Science and Technology, Taiwan [grant number MOST106-2314-B-075-031-MY], Ministry of Health and Welfare, Taiwan [grant number MOHW110-TDU-B-211-144019], and Taipei Veterans General Hospital, Taiwan [grant number V110C-106, V109E-007-2(110) and V111E-001-2].

## Conflict of Interest

C-LC had received honoraria from AstraZeneca, Boehringer Ingelheim, and Roche. C-IS had received honoraria from Boehringer Ingelheim. -CHC had received honoraria from AstraZeneca, Boehringer Ingelheim, Pfizer, and Roche.

The remaining authors declare that the research was conducted in the absence of any commercial or financial relationships that could be construed as a potential conflict of interest.

## Publisher’s Note

All claims expressed in this article are solely those of the authors and do not necessarily represent those of their affiliated organizations, or those of the publisher, the editors and the reviewers. Any product that may be evaluated in this article, or claim that may be made by its manufacturer, is not guaranteed or endorsed by the publisher.
